# Supplementary material for: Biopolymer Composites as an Alternative to Materials for the Production of Ecological Packaging
Source: Polymers (Basel). 2021 Feb 16;13(4):592. doi: 10.3390/polym13040592 (PMC7920263; doi:10.3390/polym13040592)
Supplement: Supplementary file 1 [file polymers-13-00592-s001.pdf]

polymers

MDPI

Suplementary

Figure in Supplementary

Figure *S1.* Pictures of gelatin-based base compositions (private photo resources, CanoScan 4400F, Canon, Tokyo, Japan).

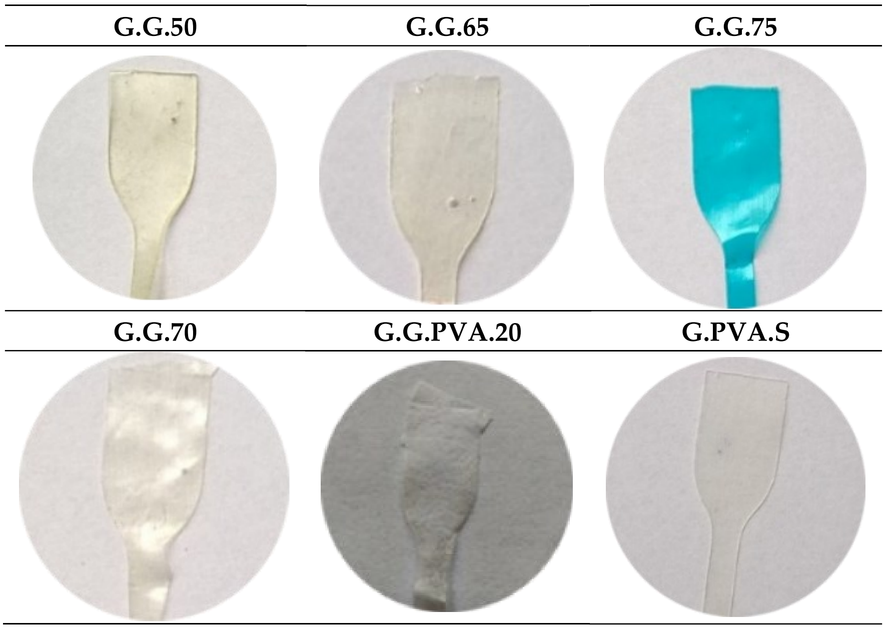

Table S1. Characterization of the functional groups of gelatin composites using the infrared medium and long range FTIR.

Composites	IR ν_{max}[cm^{-1}]									
	**ν(O−H)**	**ν(N−H)**	**ν(C-H)**	**ν(-C=O)**	**ν(N-H)**	**ν(C-OH)**	**ν(-C-N-)**	**ν(-O-C-O)**	**ν(C=C)**	**ν(PO_4^{3-})**
G.G.75	3560	3284	2914/2926	1630	1535	1030	1215			
G.G.S.75	3663	3275	2934/2976	1628	1545	1027	1229			
G.G.C.75	3651	3286	2922/2876	1661	1535	1231		1405		
G.G.PVA.75	3286		2927/2875		1537	1029	1230		1627	
G.G.P.75	3275	3275	2925/2825	1628	1535	1030	1237			1030
